# Accessibility of Low-cost Insulin From Illegitimate Internet Pharmacies: Cross-sectional Study

**DOI:** 10.2196/25855

**Published:** 2022-02-14

**Authors:** Benjamin Penley, Lana Minshew, Hui-Han Chen, Stephen Eckel, Sachiko Ozawa

**Affiliations:** 1 Division of Practice Advancement and Clinical Education Eshelman School of Pharmacy University of North Carolina at Chapel Hill Chapel Hill, NC United States; 2 Center for Innovative Pharmacy Education and Research Eshelman School of Pharmacy University of North Carolina at Chapel Hill Chapel Hill, NC United States; 3 Department of Maternal and Child Health Gillings School of Global Public Health University of North Carolina at Chapel Hill Chapel Hill, NC United States

**Keywords:** insulin, diabetes, internet, online, pharmacy, medication, cost

## Abstract

**Background:**

There is much public debate regarding the high cost of insulin. With 1-in-4 patients in the United States with type 1 diabetes reporting difficulties affording insulin, there is concern that some of these patients might look for cost savings on the internet, unaware that 96% of internet pharmacies are illegitimate. Patients who purchase insulin from illegitimate internet pharmacies remove themselves from traditional health care systems that ensure safe, quality-assured, and effective medication use.

**Objective:**

This study aims to determine the accessibility of Humalog and NovoLog insulin from internet pharmacies and characterize how these sites approached patient safety, and priced as well as marketed their products.

**Methods:**

From September to December 2019, we queried the phrases *buy insulin online, buy Humalog online*, and *buy NovoLog online* in common search engines. The first 100 search results from Google and Bing, and the first 50 search results from Yahoo! and DuckDuckGo were screened. Websites were included if they claimed to sell Humalog or NovoLog insulin, were active, free access, in the English language, and had a unique URL. The legitimacy of websites was classified using LegitScript. Safety and marketing characteristics were compared across the legitimacy of internet pharmacies. Internet pharmacy prices were compared with the prices offered through brick-and-mortar pharmacies using GoodRx.

**Results:**

We found that 59% (n=29) of the 49 internet pharmacies in our analysis were illegitimate, whereas only 14% (n=7) were legitimate and 27% (n=13) were unclassified. Across illegitimate internet pharmacies, Humalog and NovoLog insulin were 2 to 5 times cheaper as compared with both legitimate internet pharmacies and brick-and-mortar stores. Risks associated with the use of illegitimate internet pharmacies by American consumers were evident: 57% (8/14) did not require a prescription, 43% (6/14) did not display medication information or warnings, and only 21% (3/14) offered access to purported pharmacists. This included 9 rogue internet pharmacies that sold Humalog and NovoLog insulin within the United States, where 11% (1/9) required a prescription, 11% (1/9) placed quantity limits per purchase, and none offered pharmacist services. Rogue internet pharmacies often offered bulk discounts (11/18, 61%), assured privacy (14/18, 78%), and promoted other products alongside insulin (13/18, 72%). The marketing language of illegitimate internet pharmacies appealed more to quality, safety, and customer service as compared with legitimate sites.

**Conclusions:**

The ease of access to low-cost insulin through illegitimate internet pharmacies calls for urgent attention. Illegitimate internet pharmacies place patients at risk of poor-quality medications and subpar pharmacy services, resulting in adverse events and poor diabetes control. A multifaceted approach is needed to close illegitimate internet pharmacies through legal and regulatory measures, develop better search engine filters, raise public awareness of the dangers of illegitimate internet pharmacies, and address the high costs of insulin.

## Introduction

### Access to Insulin

For patients with type 1 or type 2 advanced diabetes, insulin is the cornerstone of therapy. Furthermore, 1 in 10 Americans (around 34.2 million people) have diabetes, with nearly 1.6 million living with type 1 diabetes [1]. Patients with type 1 diabetes cannot produce endogenous insulin and thus require treatment with exogenous insulin. For patients living with type 2 diabetes, insulin is often required if adequate glycemic control is not maintained with lifestyle modifications and noninsulin medications. In patients who require insulin, regulation of blood glucose is tantamount to disease control; if uncontrolled, it can result in acute, life-threatening conditions of diabetic ketoacidosis or severe hypoglycemia, as well as chronic but still life-threatening complications such as cardiovascular disease, nephropathies, retinopathies, and neuropathies [2]. These complications can result in—among other outcomes—dialysis-dependence, blindness, amputations, serious quality-of-life reductions, and death [2].

Increasingly, rising costs unique to the US market have hindered access to insulin for patients [3]. Despite legislative initiatives to control prescription drug costs, high insulin costs in the United States persist, with list prices of insulin tripling between 2003 and 2013 [3]. Although insulin was first used as a medication in 1922, the insulin market remains dominated by branded products, with no actual generic drug approved. This is important as generic competition has been shown to decrease medicine prices by 60% on average, when 3 or more manufacturers that are generic are in the market [4]. High costs are associated with all insulin types, but rapid-acting insulin analogs such as Humalog (insulin lispro) and NovoLog (insulin aspart) are among the highest priced [3]. Humalog’s list price, for example, continued to increase after 2014 from US $391 to nearly US $600 in 2017—a list price increase similar to that of its competitor NovoLog. It is worth noting that net prices remained relatively stable for Humalog and NovoLog during that time frame, meaning that an increase in costs was primarily offset by discounts made available [5]. Regardless, high list prices have a direct impact on patients; in 2019, 1 in 4 patients in the United States with type 1 diabetes reported difficulties affording their medication [6]. Patients such as these are often led to make difficult decisions. More than 1 in 4 patients in the United States with type 1 diabetes reported rationing their insulin in 2019 [7]. Patients who struggle to access insulin from traditional methods, namely brick-and-mortar pharmacies, might look for alternative, lower-cost methods, such as purchasing insulin from friends, across borders, or from illegitimate internet pharmacies [8,9].

### Growth of Internet Pharmacies

Internet pharmacies are a popular destination for the purchase of prescription drugs, with 30,000 to 35,000 internet pharmacies accessible in 2016 [10]. Internet pharmacies are defined by whether they operate as legitimate pharmacies or whether they are illegitimate and in violation of US pharmacy laws and practice standards [11]. It has been reported that 96% of all accessible internet pharmacies are illegitimate [10]. According to the World Health Organization, more than 50% of medications acquired from internet pharmacies that do not advertise their physical location (a common characteristic of illegitimate internet pharmacies) are counterfeit or substandard [12]. The dangers associated with illegitimate internet pharmacies have led to the implementation of rules and regulations to ensure safe internet pharmacy use. For example, several states in the United States require internet pharmacies to be accredited with the National Association of Boards of Pharmacy (NABP) to receive licensure [13]. However, the enforcement of such rules and regulations is complicated by an intricate e-commerce environment composed of numerous, often international, stakeholders [11]. The complexity and anonymity of e-commerce allows illegitimate internet pharmacies to avoid detection, and even when detected, reopen operations under new web addresses [14]. Given the evasiveness and high prevalence of illegitimate internet pharmacies, there is concern that patients purchasing medications on the internet might be subject to low-quality products, which could result in the development of dangerous adverse effects, especially for high-risk medications such as insulin.

Beyond concerns related to medication quality, there is also concern regarding the lack of services offered by illegitimate internet pharmacies [15]. In choosing illegitimate internet pharmacies, patients opt out of medication counseling, monitoring, and drug-drug interactions checking that pharmacists and other health care professionals provide to ensure proper medication use [16]. The use of these resources is well documented to improve patient outcomes [17]. Insufficient safety measures could also be further exacerbated by marketing methods that illegitimate internet pharmacies use to attract consumers. Although it has been shown that patients using illegitimate internet pharmacies are at greater risk of developing adverse effects from treatment, there is a lack of current data on how illegitimate internet pharmacies approach patient safety and the marketing methods they use, particularly for high-risk medications [18].

### Objective

The accessibility of rapid-acting insulins from illegitimate internet pharmacies could pose a threat to patient safety. We investigated the availability of Humalog and NovoLog insulin from internet pharmacies through common search engines and documented the website’s safety and marketing characteristics, as well as the costs of Humalog and NovoLog insulin.

## Methods

### Overview

Humalog and NovoLog were chosen for our analysis because of their relatively recent approval by the US Food and Drug Administration (FDA), high list prices, and their outsized role in the discussion of medication pricing in the United States [3]. Website screening was conducted from September to December 2019 using 4 search engines (Google, Bing, Yahoo!, and DuckDuckGo) with the phrases *buy insulin online*, *buy Humalog online*, and *buy NovoLog online*. For each search phrase, the first 100 results from Google and Bing and the first 50 of Yahoo! and DuckDuckGo were screened. Google, Bing, and Yahoo! were chosen for their widespread use in the United States. In addition, DuckDuckGo was searched because of its emphasis on user privacy. Websites were included if they claimed to sell Humalog or NovoLog insulin, were active, free access, in the English language, and had a unique URL. Websites selling Humalog or NovoLog insulin that were accessed from search engine results through a landing page were included. Screenshots were taken of each website for records.

The legitimacy of websites was assessed using LegitScript, which classifies pharmacies according to licensure or registration in affiliated jurisdictions, sale of controlled substances, previous discipline, requirement of valid prescription, protection of privacy, patient services offered, transparency, and domain name registration [19]. LegitScript was chosen to assess internet pharmacy legitimacy because of the breadth of internet pharmacies that it monitors (81,000+) and its partnerships with private (eg, Google, Amazon, Facebook) and governmental agencies (eg, FDA). The websites in this analysis were classified as (1) illegitimate, subclassified as (1a) rogue—*these merchants engage in illegal, unsafe, or misleading activities such as selling prescription drugs without a prescription* and (1b) unapproved—*there is some problem with regulatory compliance or risk, but it is typically less egregious than rogue* or (2) legitimate—*these merchants are registered with a LegitScript certification program and have passed LegitScript certification criteria* or (3) unclassified—no information was available from LegitScript.

The average monthly traffic to website domains defined as unique visits from any country was obtained from SimilarWeb [20]. This website aggregates information on website traffic from a variety of sources. We compared website traffic with the legitimacy of internet pharmacies. The IP addresses of internet pharmacies were examined using IP2location, which retrieves geographic information based on IP addresses [21]. IP address locations were compared with their listed locations.

### Costs

Costs of Humalog and NovoLog 100 IU/mL insulin at the most frequently sold dosage forms (ie, 1×10-mL vials, 5×3-mL pens, 5×3-mL cartridges) were collected from a subset of internet pharmacies offering to ship within the United States. Prices per mL of 100 IU/mL Humalog and NovoLog insulin for vials, pens, and cartridges were calculated. The shipping costs and bulk discounts were not considered in the cost calculations. Internet pharmacy prices were compared with prices offered through GoodRx, a drug coupon website. GoodRx prices are representative of out-of-pocket prices that uninsured US patients might pay at brick-and-mortar stores. Average prices at brick-and-mortar stores were obtained from GoodRx on April 30, 2020. Costs were averaged by legitimacy of internet pharmacies among websites selling Humalog and NovoLog pens that offered US shipping.

### Marketing

Marketing characteristics were selected based on previous literature, and the analysis focused on internet pharmacies classified by LegitScript [11,16,22,23]. Marketing characteristics were compared across the legitimacy of internet pharmacies. Cost-related marketing characteristics included whether internet pharmacies offered bulk discounts or promo codes. Promotional marketing characteristics included whether internet pharmacies displayed specific medication advertisements pertaining to any form of insulin, advertisements for other products on the page advertising Humalog or NovoLog insulin, or customer testimonies. Marketing characteristics appealing to customer service and general reputability included whether internet pharmacies displayed a phone number, offered the assistance of an associate, or claimed pharmacy registration in some form (eg, Canadian International Pharmacy Association, International Pharmacy Association of British Columbia, or PharmacyChecker [24]). Additional marketing characteristics included whether internet pharmacies offered privacy assurances (eg, discrete packaging or protection of health- or billing-related information) or offered shipping within the United States.

We qualitatively analyzed the website marketing language by collecting texts from the homepages of all included internet pharmacies. After initially reading through the texts to identify the most common marketing language, we selected and defined 6 characteristics. Texts were then screened to assess whether these six characteristics were discussed: (1) quality, (2) safety, (3) customer service, (4) reputability, (5) affordability, and (6) convenience. Quotes representative of each characteristic were collected by legitimacy of internet pharmacies.

### Safety

Safety characteristics were selected based on previous literature and focused on internet pharmacies classified by LegitScript [11,16,22,23]. To allow a specific focus on US pharmacies, or those that should be compliant with US laws and regulations, safety characteristics were analyzed only for internet pharmacies that offered shipping within the United States. Safety characteristics were compared across the legitimacy of internet pharmacies. Basic pharmacy-related characteristics included the requirement of a prescription and controls on the amount of Humalog or NovoLog insulin that could be ordered (eg, restricting patients to a 90-day supply or the quantity listed on their prescription). Characteristics related to pharmacy services included whether there was an offer to speak with a pharmacist and whether medication information and drug-related warnings and precautions were displayed on the product page. Characteristics related to location included whether the pharmacy listed a physical location and the website location listed matched the country of the IP address.

## Results

### Overview

We screened 300 websites and identified a total of 49 internet pharmacies that claimed to sell Humalog or NovoLog insulin. Of the internet pharmacies, LegitScript classified 59% (29/49) as illegitimate, including 37% (18/49) as rogue and 22% (11/49) as unapproved, whereas 14% (7/49) were legitimate and 27% (13/49) were unclassified. The listed locations of these internet pharmacies differed, with 41% (12/29, all rogue sites) of illegitimate internet pharmacies advertising no location. Of the 29 illegitimate internet pharmacies, 52% (15, 4 rogue and 11 unapproved sites) advertised a Canadian location. The remaining illegitimate internet pharmacies claimed to be located in Great Britain (1/29, 3%) or in Europe (1/29, 3%). No illegitimate internet pharmacies advertised a US location. For legitimate internet pharmacies, 43% (3/7) advertised locations in the United States, 29% (2/7) in Australia, 14% (1/7) in Canada, and 14% (1/7) in India. The majority (4/7, 57%) of legitimate internet pharmacies’ physical locations listed on their websites matched those of their server locations. In contrast, physical and server locations matched only 27% (3/11) of the time among unapproved internet pharmacies. None of the physical and server locations matched among the 18 rogue internet pharmacies.

Traffic to internet pharmacies, as determined by SimilarWeb [20], differed depending on the legitimacy of internet pharmacies. Although illegitimate internet pharmacies were the most abundant in the search results, unique monthly visits to each site were comparatively lower for illegitimate internet pharmacies (0-250,000) than to legitimate internet pharmacies (5000-63.6 million). The 3 US-based legitimate internet pharmacies received the highest volumes of unique monthly traffic reported at 63.6 million (Costco), 40.35 million (CVS), and 1.17 million (Healthwarehouse) visits per month [25-27].

### Costs

The costs of Humalog and NovoLog insulin vials, pens, and cartridges were recorded only for internet pharmacies shipping within the United States (n=22). The most commonly sold volume and strength of the insulin vials were 10 mL at 100 IU/mL. Humalog and NovoLog insulin 3 mL pens and 3 mL cartridges were most often sold in packages containing a quantity of 5, which is in alignment with product packaging available from brick-and-mortar pharmacies. The cost per mL of 100 IU/mL insulin varied depending on the legitimacy of the internet pharmacies. For rogue internet pharmacies, the average costs of insulin varied depending on dosage forms: Humalog vials cost US $11.30 (n=1), Humalog pens cost US $7.84 (n=4), and Humalog cartridges cost US $10.04 (n=4), whereas NovoLog vials cost US $5.90 (n=2), NovoLog pens cost US $8.47 (n=7), and NovoLog cartridges cost US $8.00 (n=3). Costs were similarly low in unapproved and unclassified internet pharmacies.

**Figure 1:**
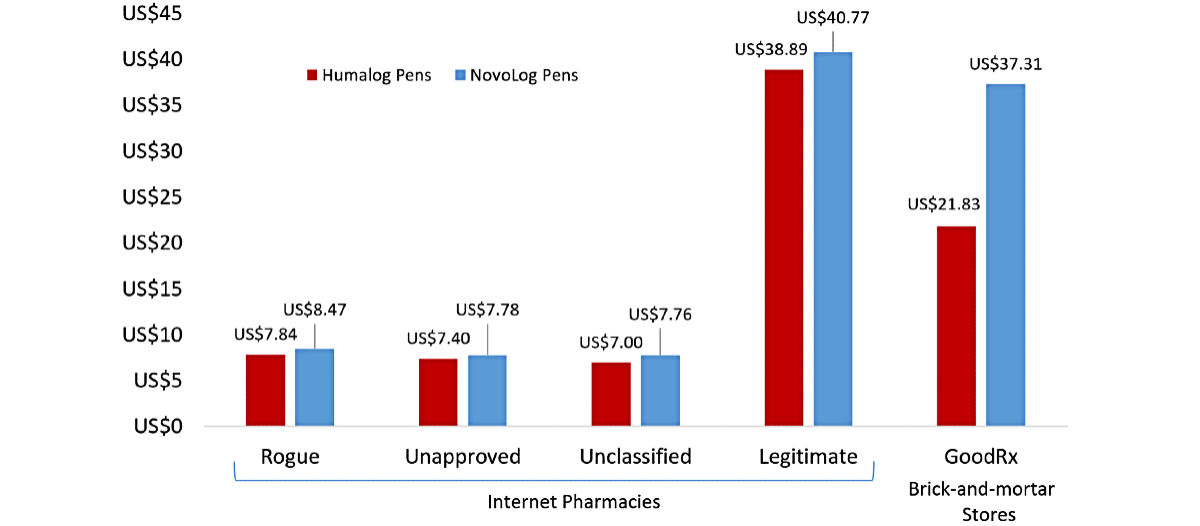
Costs of Humalog and NovoLog insulin pens available in the United States on the internet and at brick-and-mortar stores.

For legitimate internet pharmacies, the average costs of insulin (without insurance) were two- to five-fold higher: Humalog vials cost US $31.51 (n=2) and Humalog pens cost US $38.89 (n=2), whereas Humalog cartridges were not available; meanwhile NovoLog vials cost US $32.77 (n=2), NovoLog pens cost US $40.77 (n=2), and NovoLog cartridges cost US $38.47 (n=2). Compared with illegitimate pharmacies, GoodRx costs—representative of average costs at brick-and-mortar pharmacies—were also approximately 2 to 5 times more expensive. However, compared with the cost of legitimate internet pharmacies for uninsured patients excluding shipping costs, GoodRx prices were marginally cheaper: Humalog vials cost US $17.22, Humalog pens cost US $21.83, and Humalog cartridges cost US $34.13, whereas NovoLog vials cost US $29.38, NovoLog pens cost US $37.31, and NovoLog cartridges cost US $35.90. The difference in costs per mL of Humalog and NovoLog insulin pens (the most common dosage form in our analysis) depending on the source is depicted in Figure 1.

### Marketing

The marketing characteristics of internet pharmacies selling Humalog and NovoLog insulin and classified by LegitScript (N=36) are described in Table 1. Rogue internet pharmacies differed from unapproved and legitimate internet pharmacies across several characteristics. More often, rogue internet pharmacies offered bulk discounts (11/18, 61%), assured privacy (14/18, 78%), and promoted other products on the Humalog or NovoLog insulin product pages (13/18, 72%). Although rogue internet pharmacies offered some form of contact through email or chat functions, most sites did not offer a phone number (11/18, 61%). Both legitimate (n=7) and unapproved (n=11) internet pharmacies shared similar characteristics, where few offered bulk discounts (2/7, 29% legitimate; 2/11, 18% unapproved), all displayed a phone number (18/18, 100%), and most touted registration or made accreditation claims (6/7, 86% legitimate; 10/11, 91% unapproved).

**Table 1:** Marketing characteristics of internet pharmacies selling Humalog or NovoLog^a^.

Characteristics	Rogue (n=18), n (%)				Unapproved (n=11), n (%)				Legitimate (n=7), n (%)		
	Yes	No	Not reported	Yes		No	Not reported	Yes		No	Not reported
US shipping of insulin	9 (50)	9 (50)	0 (0)	5 (45)		6 (55)	0 (0)	3 (43)		4 (57)	0 (0)
Bulk discounts	11 (61)	5 (28)	2 (11)	2 (18)		8 (73)	1 (9)	2 (29)		5 (71)	0 (0)
Coupons	9 (50)	8 (44)	1 (6)	2 (18)		9 (82)	0 (0)	4 (57)		3 (43)	0 (0)
Registration claims	8 (44)	10 (56)	0 (0)	10 (91)		1 (9)	0 (0)	6 (86)		1 (14)	0 (0)
Privacy assurances	14 (78)	4 (22)	0 (0)	8 (73)		3 (27)	0 (0)	1 (14)		6 (86)	0 (0)
Customer testimonies	10 (56)	8 (44)	0 (0)	7 (64)		4 (36)	0 (0)	3 (43)		4 (57)	0 (0)
Offer to speak with associate	18 (100)	0 (0)	0 (0)	9 (82)		2 (18)	0 (0)	6 (86)		1 (14)	0 (0)
Phone number	7 (39)	11 (61)	0 (0)	11 (100)		0 (0)	0 (0)	7 (100)		0 (0)	0 (0)
Insulin-specific advertisements	0 (0)	18 (100)	0 (0)	3 (27)		8 (73)	0 (0)	0 (0)		7 (100)	0 (0)
Advertisements for other products on page selling insulin	13 (72)	5 (28)	0 (0)	1 (9)		10 (91)	0 (0)	4 (57)		1 (14)	2 (29)

^a^Illustrates marketing characteristics of 36 websites selling Humalog or NovoLog. This table does not include 13 internet pharmacies selling Humalog or NovoLog insulin that were not classified by LegitScript.

Marketing language from website homepages differed according to the legitimacy of internet pharmacies, particularly for quality, safety, and customer service. Although 83% (24/29) of illegitimate internet pharmacies appealed to quality, only 29% (2/7) of legitimate internet pharmacies used language that suggest the quality of medication or services. Marketing language appealing to safety (19/29, 66% illegitimate vs 1/7, 14% legitimate) and customer service (24/29, 83% vs 4/7, 57%) were also more common among illegitimate internet pharmacies. The frequency of use of marketing language was similar among reputability (16/29, 55% vs 4/7, 57%), affordability (24/29, 83% vs 5/7, 71%), and convenience (22/29, 76% vs 5/7, 71%). Differences in marketing language are also demonstrated through the selection of quotes in Table 2. The marketing language of illegitimate internet pharmacies tended to communicate a sense of urgency to purchase products and strongly emphasized the merits of the pharmacy. For example, one quote from an illegitimate internet pharmacy appealed to reputation, affordability, and safety:

If you are looking to buy your prescription drugs in Canada, through a reputable international or online Canadian pharmacy, [our online pharmacy] provides you access to a trusted source of affordable and safe prescription drugs.

Illegitimate pharmacy

**Table 2:** Types of marketing language used on the home pages of internet pharmacies.

Characteristics	Description	Illegitimate (n=29)		Legitimate (n=7)	
		Values, n (%)	Selected quote^a^	Values, n (%)	Selected quote
Quality	Language suggesting quality of medication or services	24 (83)	“We guarantee that all [medications] for sale on this site are 100% genuine and extremely powerful.”	2 (29)	“[We] offer you quality care...”
Safety	Language explicitly referring to the safety of medication products, internet ordering platform, or other services	19 (66)	“When it comes to your health, we know that safety is your number one concern. It’s ours too.”	1 (14)	“Besides delivering medicines at your doorstep, we...help people use their medicines effectively and safely.”
Customer service	Language suggesting availability of staff to answer questions or remedy problems	24 (83)	“24/7 customer support (we are always at your disposal!).”	4 (57)	“Customer service: get answers to your questions.”
Reputability	Language suggesting renown in selling or accreditation to sell prescription drugs	16 (55)	“[Our pharmacy] has a great reputation serving the community for 47 years and counting.”	4 (57)	“Accredited and Certified in all 50 states.”
Affordability	Language suggesting discounts or cheap prescription drugs	24 (83)	“[We] provide the same insulin that’s available in the US except our prices are much lower, and we pass on the savings to you.”	5 (71)	“At [our pharmacy], you can buy health products and medicines online at best discounts.”
Convenience	Language suggesting ease of internet pharmacy use or prescription drug purchase; fast delivery or services that allow for time savings	22 (76)	“The process of payment through Bitcoin is simple. You need to go through only a few steps to quickly confirm and complete your order.”	5 (71)	“You get the convenience of online shopping combined with the support and guidance of our dedicated team.”

^a^Quotes were taken directly from internet pharmacy websites.

### Safety

The safety characteristics of internet pharmacies selling Humalog and NovoLog insulin within the United States and classified by LegitScript (N=17) are described in Table 3. Overall, illegitimate internet pharmacies revealed poor patient safety records: 57% (8/14) did not require a prescription, 43% (6/14) did not display medication information or warnings, and only 21% (3/14) offered access to pharmacists. Rogue internet pharmacies differed from legitimate internet pharmacies more substantially than unapproved internet pharmacies in terms of patient safety. Rogue internet pharmacies seldom required a prescription (1/9, 11%) or placed quantity limits on the amount of medication that could be ordered (1/9, 11%), and none offered pharmacist services (0/9, 0%). Unapproved internet pharmacies uniformly claimed to require a prescription (5/5, 100%) and placed quantity limits (5/5, 100%), and some sites offered pharmacist services (3/5, 60%). Drug-related information and warnings were not uniformly displayed for both rogue and unapproved internet pharmacies.

Data were unavailable for one legitimate internet pharmacy regarding whether pharmacist services were offered (where member registration was required). Not all legitimate internet pharmacies were accredited through the NABP because of geography. However, legitimate internet pharmacies required or displayed characteristics consistent with best internet pharmacy communication practices, such as requiring pharmacists to offer individual, meaningful consultations [13].

**Table 3:** Safety characteristics of internet pharmacies selling Humalog or NovoLog insulin in the United States.

Safety characteristics	Rogue (n=9), n (%)				Unapproved (n=5), n (%)				Legitimate (n=3), n (%)		
	Yes	No	Not reported	Yes		No	Not reported	Yes		No	Not reported
Prescription required	1 (11)	8 (89)	0 (0)	5 (100)		0 (0)	0 (0)	3 (100)		0 (0)	0 (0)
Offer to speak with pharmacist	0 (0)	9 (100)	0 (0)	3 (60)		2 (40)	0 (0)	2 (67)		0 (0)	1 (33)^a^
Medication precautions on product page	6 (67)	3 (33)	0 (0)	2 (40)		3 (60)	0 (0)	3 (100)		0 (0)	0 (0)
Medication information on product page	6 (67)	3 (33)	0 (0)	2 (40)		3 (60)	0 (0)	3 (100)		0 (0)	0 (0)
Quantity control	1 (11)	8 (89)	0 (0)	5 (100)		0 (0)	0 (0)	3 (100)		0 (0)	0 (0)
Lists a physical location	4 (44)	5 (56)	0 (0)	5 (100)		0 (0)	0 (0)	3 (100)		0 (0)	0 (0)
Location listed on website and location of server match	0 (0)	9 (100)	0 (0)	2 (40)		3 (60)	0 (0)	3 (100)		0 (0)	0 (0)

^a^One legitimate pharmacy required an account to gain access to services. Overall, 5 internet pharmacies selling Humalog or NovoLog insulin in the United States were not classified using LegitScript.

## Discussion

### Principal Findings

Our analysis demonstrates that both Humalog and NovoLog insulin are readily available from internet pharmacies that engage in illegal sales of prescription drugs. Illegitimate internet pharmacies were found to be abundant using common search engines, outnumbering legitimate internet pharmacies. Of the internet pharmacies included in our analysis, nearly 60% (29/49) were illegitimate, whereas only 14% (7/49) were legitimate (with the remainder unclassified). The widespread availability of illegitimate internet pharmacies poses a threat to unsuspecting consumers and provides easy access to those seeking insulin without a prescription [9]. Incentives to purchase insulin from illegitimate internet pharmacies go beyond ease of access, as our analysis reveals that these internet pharmacies offer substantial price reductions as compared with brick-and-mortar stores and legitimate internet pharmacies.

### Rising Insulin Costs

In the United States, rising list prices on rapid-acting insulin analogs such as Humalog and NovoLog insulin have resulted in a substantial cost burden for patients with diabetes. Legislative initiatives to curb insulin costs include, among others, setting out-of-pocket maximums and allowing personal drug importation. However, these reforms have only been trialed in some states, and the legality of the personal importation of insulin remains contentious [28,29]. In 2018, in the midst of public outcry at high insulin costs, the manufacturer Eli Lilly introduced Lispro, an authorized generic to Humalog, at a 50% discount on the Humalog list price [30]. Unfortunately, according to a Senate report, the uptake of Lispro has been meager at best, with 83% of 386 surveyed national pharmacies not having Lispro in stock and 69% unable to order the medication [31]. In 2020, the Trump Administration announced that Medicare would begin offering 1750 different insurance plans that capped out-of-pocket spending to US $35 for insulin [32]. However, this out-of-pocket maximum is limited only to Medicare beneficiaries. Given the relatively limited action from the federal government, manufacturers, and other key players in the United States pharmaceutical supply chain, high insulin list prices and out-of-pocket costs for many persist. Many patients continue to face insulin access problems, particularly those who are uninsured, which in 2018 accounted for 8.5% of the US population [3,33].

With financial pressure, some patients who need insulin to manage their diabetes have resorted to illegal activities such as borrowing insulin, importing insulin from lower-cost countries, or purchasing insulin from illegitimate internet pharmacies [8]. Our analysis, which focuses on the internet pharmacy marketplace, demonstrates that among pharmacies that ship within the United States, the per mL cost of Humalog and NovoLog insulin from illegitimate internet pharmacies was approximately 2 to 5 times cheaper than that offered by legitimate internet pharmacies or GoodRx. Such substantial price differences raise concerns that illegitimate internet pharmacies may appeal to patients priced out of traditional means of acquiring insulin. Beyond offering lower prices, rogue internet pharmacies do not require prescriptions and also use marketing methods that appeal to cost-conscious consumers, such as offering bulk discounts or coupons. In addition, illegitimate pharmacies appeal to affordability through language on their homepages.

Although costs are lower for Humalog and NovoLog insulin from illegitimate internet pharmacies, they remain illegal because of the serious risks associated with their use. Humalog and NovoLog insulin are high-risk medications that require both therapeutic monitoring to ensure optimal short- and long-term outcomes and sufficient counseling for the prevention of adverse events such as hypoglycemia. Between 2007 and 2009, nearly 20% of emergency hospitalizations for the treatment of emergent adverse events were because of insulin [34]. The costs of these visits are not trivial; in 2016, the total average cost per person per visit for hypoglycemia was US $1965 for an emergency department visit and US $11,632 for inpatient hospitalization in the United States [35]. The American Diabetes Association recommends that patients treated with insulin who are unaware of hypoglycemia should be counseled on signs and methods to treat it [36].

### Threat of Illegitimate Internet Pharmacies

Our analysis demonstrates that illegitimate internet pharmacies, particularly rogue internet pharmacies, do not offer pharmacy services that are on-par with those offered through legitimate internet pharmacies. The majority (8/9, 89%) of rogue internet pharmacies allowed the purchase of Humalog or NovoLog insulin in the United States without a prescription, precluding the involvement of health care professionals in patient care. Further preventing communication with health care professionals, no rogue internet pharmacies made the offer to speak with pharmacists. On illegitimate internet pharmacy websites, patients were often left without medication information and drug-related warnings and precautions. Illegitimate internet pharmacies, with rogue internet pharmacies being the worst offenders, allow patients to access Humalog and NovoLog insulin with minimal information, predisposing these patients to poor diabetes control and potential development of adverse events.

There is also concern as to whether the quality of insulin obtained from illegitimate internet pharmacies is comparable with that obtained from legitimate internet pharmacies. Low costs offered through illegitimate internet pharmacies could suggest less stringent cold chain shipping methods or lower-quality medications. Although our analysis did not collect information on the quality of Humalog and NovoLog insulin obtained from illegitimate internet pharmacies, substandard and falsified medicines are globally prevalent [12,37]. Studies have suggested that poor medication quality from illegitimate pharmacies could further increase patient safety at risk [38]. Illegitimate internet pharmacies, as evinced in our analysis of marketing language, often make claims touting the quality of their products. Ironically, this could amplify the risk of consumers inadvertently purchasing low-quality medications. To address these risks, a multifaceted approach is needed to close illegitimate internet pharmacies, develop better search engine filters, raise public awareness of the dangers of illegitimate internet pharmacies, and address high insulin costs.

National organizations are combating the proliferation of illegitimate internet pharmacies. The FDA’s BeSafeRx campaign and the Alliance for Safe Online Pharmacies’ *Buy Safe Rx* campaign help consumers identify illegitimate internet pharmacies and recognize their risks [39,40]. The NABP helps consumers identify legitimate internet pharmacies through their list of accredited digital pharmacies, as well as with a verification service through which legitimate internet pharmacies receive a *dot-pharmacy* domain [13]. The Center for Safe Internet Pharmacies also offers a verification service through its initiative *Verify Before You Buy* (powdered by LegitScript), wherein consumers can search the URL of an internet pharmacy to check its legitimacy [41]. In May 2020, North Carolina’s Secretary of State announced a partnership with the Center for Safe Internet Pharmacies to raise consumer awareness [42].

Regulatory and legal actions are ongoing against illegitimate internet pharmacies. Operation Pangea, an effort led by Interpol in conjunction with the FDA and US Department of Justice, has resulted in the removal of thousands of illegitimate internet pharmacies [43]. LegitScript has also collaborated with the FDA to identify and close illegitimate internet pharmacies [44]. However, illegitimate internet pharmacies persist by closing and reopening under new web addresses, requiring continued vigilance by regulatory authorities.

Given that patients with diabetes are frequently counseled on the management of their disease, health care providers such as physicians and pharmacists are uniquely positioned to lead the charge in making consumers aware of the risks of acquiring insulin from illegitimate internet pharmacies. 

### Limitations

The limitations of our study include the small sample size and the cross-sectional design. However, our screening methods were consistent with what US consumers purchasing insulin on the internet might experience. An additional limitation is that we did not analyze the quality of products available from internet pharmacies, precluding conclusions pertaining to medication quality. We did not purchase medications from these websites because we questioned the ethical implications of financially supporting the operations of illegitimate internet pharmacies. Our analysis was conducted before the COVID-19 pandemic. We believe our results are even more relevant now as internet purchases have become commonplace in the United States [45]. Finally, we were unable to quantify the purchase volume of Humalog or NovoLog insulin from these internet pharmacies. The nonspecific, surrogate measure of unique monthly visits is representative of the overall traffic to these websites and is not necessarily indicative of interest in or purchases of Humalog or NovoLog insulin. Despite these limitations, we contend that our analysis reflects the internet pharmacy marketplace for insulin in the United States as accessed through common search engines.

### Conclusions

The relatively low costs of Humalog and NovoLog insulin from easily accessible illegitimate internet pharmacies place patients at risk. Although the elimination of illegitimate internet pharmacies would be the gold standard way to reduce their risk to patients, illegitimate internet pharmacies are elusive. Governmental agencies should continue to pursue legal and regulatory measures with the intent of closing illegitimate internet pharmacies. Search engines should work to filter their results better, decreasing the visibility of illegitimate internet pharmacies. Finally, although public awareness campaigns and provider-to-patient efforts can bring attention to the dangers of illegitimate internet pharmacies, they do not address the reasons that patients may visit these sites. With patient safety in mind, US legislators and members of the pharmaceutical supply chain should work to lower the costs of insulin, thereby diminishing patients’ incentive to purchase from illegitimate internet pharmacies.

## Abbreviations

FDA: Food and Drug Administration
NABP: National Association of Boards of Pharmacy
